# Gut microbiota and sepsis-associated encephalopathy: pathogenesis and precision therapies

**DOI:** 10.3389/fnins.2025.1596467

**Published:** 2025-07-08

**Authors:** Na Wei, Shiyu Dai, Wei Li, Jun Zhou, Ye Chen

**Affiliations:** ^1^Department of Anesthesiology, The Affiliated Hospital, Southwest Medical University, Luzhou, Sichuan, China; ^2^Anesthesiology and Critical Care Medicine Key Laboratory of Luzhou, The Affiliated Hospital, Southwest Medical University, Luzhou, Sichuan, China; ^3^Department of Anesthesiology, Hejiang County People’s Hospital, Luzhou, Sichuan, China; ^4^Department of Traditional Chinese Medicine, The Affiliated Hospital, Southwest Medical University, Luzhou, China

**Keywords:** sepsis-associated encephalopathy, sepsis, gut microbiota, fecal microbiota transplantation, probiotics

## Abstract

Sepsis is defined as a condition of immune dysregulation in response to an infection, and sepsis-associated encephalopathy (SAE) is often the initial symptom that manifests in patients with sepsis. This condition is characterized by its high mortality rates and the potential to cause significant disability among survivors. Despite its severity, the underlying pathophysiologic mechanisms that contribute to the development of SAE are not yet fully understood. Additionally, there are no established strict diagnostic criteria or potent treatment options available for this condition. However, an increasing body of evidence suggests that an imbalance in the gut microbiota is associated with SAE, potentially through the gut-brain axis (GBA). The GBA axis refers to the bidirectional communication between the gut microbiota and the central nervous system. In this review, we discuss the changes in the gut microbiota in SAE and the mechanisms of the GBA axis, involving neural, immune, endocrine, and neurotransmitter pathways. Finally, we conclude by evaluating the preclinical and clinical evidence for fecal microbiota transplantation and probiotics in SAE. Targeting the GBA axis will be an actionable target to ameliorate the development and progression of SAE.

## 1 Introduction

Sepsis is defined as life-threatening multiorgan dysfunction due to the body’s dysregulated response to infection, and acute brain dysfunction arising from sepsis is termed sepsis-associated encephalopathy (SAE). SAE has the potential to manifest as the initial symptom of sepsis and is observed in as many as 70% of sepsis patients (Martin-Loeches et al., 2024; Manabe and Heneka, 2022; Gofton and Young, 2012). Sepsis contributes to nearly 20% of the annual global mortality, with a rate of over 20 deaths occurring per minute. SAE has been demonstrated to be correlated with elevated mortality ratios, increased consumption of intensive care unit (ICU) resources, and extended hospital lengths of stay (Kempker and Martin, 2020; Sonneville et al., 2017). Therefore, new insights into the pathogenesis of SAE are likely to furnish a novel therapeutic direction.

In the body, the gut microbiota and the brain can communicate bidirectionally via the gut-brain axis (GBA), regulating the development and function of the immune, metabolic, and nervous systems and influencing host behavior (Morais et al., 2021). Dysbiosis of the gut microbiota is not only associated with cognitive changes (Xiang et al., 2024; Zhao et al., 2025; Grabrucker et al., 2023), but also plays a key role in the progression of sepsis (Sun et al., 2023). Gut microbiota dysbiosis increases the risk of sepsis and death, while germ-free mouse models of sepsis showed a high pathogen burden and death rate (Liang et al., 2022). Therefore, understanding the relationship between gut microbiota and SAE is crucial for further understanding the pathogenesis and treatment of SAE.

In this review, we provide an in-depth overview of the alterations in gut microbiota in sepsis and SAE exposure. We also elaborate on the impacts of gut microbiota on multiple aspects, such as impairment of barrier function, disruption of the neuroendocrine system, facilitation of neuroinflammation, induction of metabolic dysregulation, and interference with neurotransmitter function. Furthermore, we engage in a detailed discussion of targeted therapeutic approaches aimed at modulating the gut microbiota.

## 2 Dysbiosis of the gut microbiota and SAE

Numerous studies have demonstrated that gut microbiota exhibits a remarkable susceptibility to sepsis. A two-sample Mendelian randomization study presented the initial suggestive evidence of a causal link between the beneficial or deleterious impacts of gut microbiota on the risk of sepsis. The findings indicated that an augmented abundance of β – Proteobacteria, *Vibrio desulfuricans*, *Catenibacterium*, and *Hungatella* was inversely correlated with the sepsis risk. In contrast, *Clostridiaceae* 1, *Alloprevotella*, the *Lachnospiraceae* ND3007 group, and *Terrisporobacter* were potentially identified as risk factors for sepsis (Chen et al., 2023). Besides, the intestinal abundance of enteric microbiota enterotypes or enterococci could potentially function as a biomarker for predicting poor prognosis in ICU patients with sepsis (Kullberg et al., 2021). In addition, antibiotic use, host genetics, and comorbidities can also perturb the gut microbiota of sepsis. For example, the use of antibiotics in septic mice may cause intestinal microecological disorders, resulting in an increase in the inflammation-related pathogenic bacteria *Desulfovibrio* (Han et al., 2021). In two large retrospective studies, admission to the hospital for an infection-related complication and antibiotic exposure significantly increased the risk for subsequent sepsis-related hospitalization within 90 days of the index hospitalization (Baggs et al., 2018; Prescott et al., 2015). Depletion of commensal anaerobic gut microbes by anti-anaerobic antibiotics influences systemic immunity and is associated with increased mortality in patients with sepsis (Kullberg et al., 2025). These demonstrate that the progression of sepsis is associated with perturbations in the gut microbiota at both compositional and functional levels.

**FIGURE 1:**
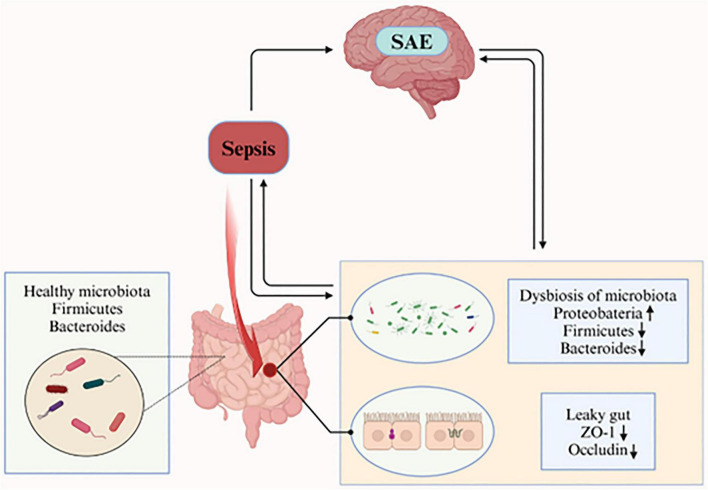
The healthy gut microbiota is dominated by the phyla Bacteroidetes and Firmicutes. During sepsis, gut microbiota composition is perturbed, characterized by increased relative abundance of Proteobacteria and decreased relative abundance of Firmicutes and Bacteroidetes. Concurrently, intestinal epithelial barrier integrity is compromised, with reduced expression of tight junction proteins ZO-1 and occludin (critical components of the epithelial barrier). These result in increased intestinal permeability (“leaky gut”), which exacerbates systemic inflammation, thereby leading to the occurrence of SAE. (Created in https://BioRender.com.)

Many studies have shown that the gut microbiota also changes during SAE in preclinical models, which is mainly characterized by a decrease in the species diversity of the gut microbiota, a decrease in the abundance of the dominant groups’ Firmicutes and Bacteroidetes, and an increase in the abundance of Proteobacteria (Table 1). Besides, the separation between sepsis and non-sepsis brain specimens as a group was driven primarily by differences in relative abundance of *Haemophilus*, *Neisseria*, and *Moraxella* species, with brain specimens of individual patients with sepsis dominated by gut-associated taxa, such as *Enterobacteriaceae* sp. and *Bacteroides* sp. (Singer et al., 2018). Additionally, studies have shown that patients with biliary tract infections and intestinal infections caused by *Staphylococcus aureus*, *Enterococcus faecium*, *Acinetobacter* spp., *Pseudomonas aeruginosa*, and *Stenotrophomonas maltophilia* were more prone to develop SAE (Zhang et al., 2012). Furthermore, another analysis identified coagulase-negative staphylococci as an independent risk factor for SAE (HR = 1.919, *P* < 0.001), but not for mortality. Methicillin-resistant *S. aureus* (MRSA) was linked to increased mortality in SAE patients (HR = 3.423, *P* < 0.001) (Fei et al., 2025). These suggest that there may be an association between gut microbiota dysbiosis and SAE, and that sepsis-induced gut microbiota dysbiosis may be involved in the etiology of SAE (Figure 1).

**TABLE 1:** Changes in bacterial flora during SAE.

Diseases	Comparison	Phylum	Observed changes	Reference
SAE	Sham vs. LPS	Proteobacteria ↑ Firmicutes ↓ Bacteroidetes ↓		Li et al., 2021
SAE	Sham vs. LPS	Proteobacteria ↑ Firmicutes ↓ Bacteroidetes ↓		
SAE	Control vs. LPS	Bacteroidota ↑ Proteobacteria ↑ Campilobacterota ↑ Actinobacteriota ↑ Patescibacteria ↑ Cyanobacteria ↑ Firmicutes ↓ Desulfobacterota ↓ Verrucomicrobiota ↓ Deferribacterota ↓	*Lachnospiraceae*-NK4A136-group ↑ *Alloprevotella* ↑ *Desulfovibrio* ↑ *Alistipes* ↑ *Bacteroides* ↑ *Muribaculum* ↑ *Helicobacter* ↑ *Colidextribacter* ↑ *Lactobacillus* ↓ *Lachnoclostridium* ↓	Xu et al., 2024
SAE			g-*Klebsiella* spp. ↑ s-Uncultured-bacterium-g-*Klebsiella* spp. ↑ *Eubacterium-coprostanoligenes* ↓ *Eubacterium-coprostanoligenes*-group ↓ *Eubacterium-hallii*-group ↓ f-Ruminococcaceae ↓	Wang H. et al., 2022
SAE	Sham vs. LPS	Proteobacteria ↑ Firmicutes ↓ Bacteroidetes ↓	*Campylobacter* ↑ *Staphylococcus* ↑ *Pseudomonas* ↑ *Bifidobacterium* ↓ *Lactobacillus* ↓ *Bacteroides* ↓ *Clostridium* ↓ *Enterobacter* ↓ *Enterococcus* ↓ *Bifidobacterium* ↓ *Lactobacillus* ↓	Li et al., 2018
Sepsis-induced cognitive decline	Sham vs. CLP	Actinobacteria ↑ Proteobacteria ↑	*Actinobacteria* ↑ *Gammaproteobacteria* ↑ *Clostridia* ↓	Giridharan et al., 2022
SAE	Sham vs. CLP		*Allobaculum* ↓ *Bacteroides* ↓ *Bifidobacterium* ↓	Liao et al., 2022
Aging SAE	Control vs. D-gal	Bacteroidota ↑ Verrucomicrobiota ↑ Firmicutes ↓ Desulfobacterota ↓	*Akkermansiaceae* ↑ *Muribaculaceae* ↑ *Akkermansia muciniphila* ↑ *Desulfovibrionaceae* ↓ *Lachnospiraceae* ↓	Pinitchun et al., 2024

LPS, lipopolysaccharides; CLP, cecal ligation and puncture.

## 3 Crosstalk between gut microbiota dysbiosis and SAE

Current studies have demonstrated that SAE is a multifactorial disease, and its pathogenesis potentially involves blood–brain barrier (BBB) disruption, neuroinflammation, microcirculatory dysfunction, neurotransmitter imbalance, and mitochondrial dysfunction (Manabe and Heneka, 2022; Pan et al., 2022; Krzyzaniak et al., 2023; Catarina et al., 2021; Huang et al., 2021; Iacobone et al., 2009). Previous studies indicate that gut microbiota exerts a significant effect on neurodevelopment and cognitive function through the GBA axis. Its abnormal changes are closely associated with many neurological disorders, especially cognitive dysfunction (Xiao et al., 2022; Sampson et al., 2016; Matheson and Holsinger, 2023). As described above, gut microbiota’s species and functions change significantly in SAE. Therefore, we reviewed the possible pathogenesis of gut microbiota dysbiosis involved in SAE by regulating the GBA axis. This review aims to offer insights for better SAE diagnosis and treatment.

### 3.1 Gut microbiota dysbiosis and barrier dysfunction

**FIGURE 2:**
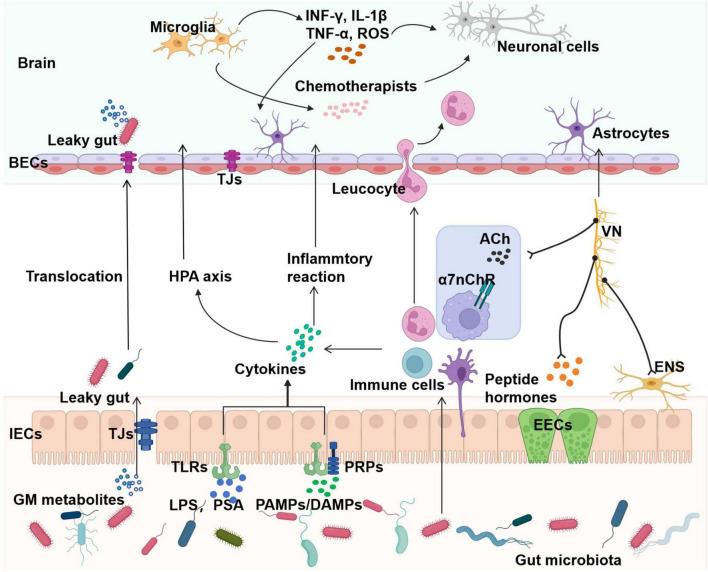
Involvement of gut microbiota in the pathogenesis of SAE. Gut microbiota dysbiosis drives SAE pathogenesis through four interconnected pathways: I. Barrier dysfunction: sepsis alters gut microbiota/metabolites, increasing intestinal and BBB permeability via reduced tight junctions (ZO-1 and occludin), allowing pathogen translocation into systemic and CNS circulation. II. Neuroendocrine axis dysregulation: gut microbiota produce hormones (e.g., serotonin) that modulate intestinal metabolism and brain function via the vagus nerve and the cholinergic anti-inflammatory pathway. III. Immune dysregulation: dysbiosis triggers systemic inflammation (TNF-α and IL-6), which crosses the BBB to activate CNS microglia/astrocytes, amplifying neuroinflammation via reactive ROS. IV. Metabolic/neurotransmitter disorders: altered microbial metabolites (e.g., SCFAs) exacerbate barrier dysfunction and disrupt neurotransmitter balance (e.g., serotonin and dopamine). Collectively, these mechanisms position the gut microbiota as a key therapeutic target for SAE, emphasizing the need for precision microbiome interventions. ROS, reactive oxygen species; BECs, cerebrovascular endothelial cells; TJs, tight junctions; HPA, hypothalamic-pituitary-adrenal; VN, vagus nerve; CAP, cholinergic anti-inflammatory pathway; IECs, intestinal epithelial cells; EECs, enteroendocrine cells; TLRs, Toll-like receptors; PRPs, pattern recognition receptors; PSA, polysaccharide A; PAMPs, pathogen-associated molecular patterns; DAMPs, danger-associated molecular patterns. (Created in https://BioRender.com.)

Loss of BBB integrity is a key cause of SAE and subsequent systemic damage (Gu et al., 2021) (Figure 2). The BBB consists of cerebrovascular endothelial cells (BECs), astrocytes, pericytes, and extracellular matrix, and this structure prevents entry of neurotoxic plasma components, blood cells, and pathogens into the brain (Obermeier et al., 2013; Sweeney et al., 2019). The BECs of the BBB are unique because of their continuous intercellular tight junctions (TJs) (Obermeier et al., 2013). Gut microbiota dysbiosis not only produces neurotoxic factors but also affects the permeability of the intestinal mucosa, leading to “leaky gut.” Inflammation caused by “leaky gut” eventually leads to “leaky brain,” i.e., increased permeability of the BBB (Lu J. et al., 2022; Hu et al., 2016). MRI imaging in SAE models reveals significant cerebral edema (Towner et al., 2018; Bozza et al., 2010). Additionally, reduced expression of the TJ proteins ZO-1 and occludin was found in brain tissues in a mouse model of SAE (Li et al., 2023). Meanwhile, disruption of the BBB is found to be associated with a loss of cerebral endothelial expression of occludin in autopsies of sepsis-related deaths (Erikson et al., 2020). Increased BBB permeability, loss of TJ proteins, and endothelial cell degeneration allow a large number of inflammatory factors and neurotoxins to enter and damage brain tissue. This process activates brain immune cells to mediate inflammatory responses, further exacerbating BBB disruption and ultimately leading to the progression of SAE (Gu et al., 2021; Sweeney et al., 2019; Lu J. et al., 2022).

### 3.2 Gut microbiota dysbiosis and neuro-endocrine disorders

The gut microbiota is involved in SAE pathogenesis through the vagus nerve. In LPS-induced SAE rat models, gut microbiota composition changed. Meanwhile, EEG activity increased, shown as higher reactivity, abnormal θ/δ rhythms, seizures, and periodic discharges. This suggests gut microbiota disruptions and cognitive problems in LPS-treated rats. Fecal microbiota transplantation (FMT) improves gut dysbiosis and cognitive function via vagus nerve-mediated mechanisms. For example, FMT in SAE rats improved gut dysbiosis, inflammation, and brain function. But after vagotomy, this improvement reversed. Additionally, studies have shown that FMT inhibits hippocampal inflammation mediator release and microglial activation via the vagus nerve, alleviating SAE-related nerve issues (Li et al., 2018). Another investigation demonstrated that intestinal dysbiosis occurring in the post-sepsis period precipitates red light exposure-induced cognitive impairment and anxiety-mimicking behaviors, with the underlying mechanism involving subdiaphragmatic vagal nerve signaling pathways (Xie et al., 2020).

Impaired neurotransmission in SAE is most commonly characterized by dysregulation of cholinergic pathways, resulting in acetylcholine deficiency and delirium-like symptoms (Krzyzaniak et al., 2023). Vagal afferents, in response to inflammation, activate vagal efferent nerves through a mechanism known as the cholinergic anti-inflammatory pathway (CAP). The CAP is an important pathway by which gut microbiota affects brain function. This cholinergic pathway is mediated primarily by nicotinic-type acetylcholine receptors on tissue macrophages, which inhibit the production of tumor necrosis factor (TNF)-α by macrophages (Wang H. et al., 2022; Andersson, 2005; Bonaz et al., 2018). It has been shown that electrical stimulation of the vagus nerve triggers anti-inflammatory effects through cholinergic pathways, improves brain function, and inhibits SAE by attenuating systemic inflammatory responses and neuroinflammation (Li et al., 2015).

In addition, a specialized sensory enteroendocrine cell (EEC) exists in the intestinal epithelium. The gut microbiota and bacterial products can bind to a series of receptors expressed by EECs to induce the release of peptide hormones, including enteric glucagon hormones (glucagon-like peptide-1 and gastric inhibitory peptide), neuropeptides (cholecystokinin and peptide YY), and 5-hydroxytryptophan (5-HT), which can act locally on intestinal neurons and mediate the regulation of gut metabolism by the GBA axis via vagal afferent pathways (Wang et al., 2020; Lach et al., 2018; Parker et al., 2020). It was found that the combination of Ghrelin and growth hormone (GH) treatment significantly inhibited the upregulation of transforming growth factor (TGF)-β expression in septic rats, and Ghrelin administration alone also reduced the levels of TNF-α and interleukin (IL)-6 in the plasma and peritoneal fluid of septic rats, and the vagotomy attenuated this beneficial effect. Whereas *in vitro* experiments, the administration of Ghrelin alone or in combination with GH did not ameliorate the levels of inflammatory factors in the LPS-induced cells (Zhou et al., 2020; Wu et al., 2007). The discrepancy between *in vivo* and *in vitro* may be related to the activation of the microbiota-neuro-endocrine axis during *in vivo* action, while the *in vitro* environment has limited ecological complexity and lacks a synergistic interaction mechanism.

Taken together, these suggest that the vagus nerve and the body’s endocrine system are involved in the information network composition of the GBA axis, and gut microbiota dysbiosis may contribute to SAE development via the GBA axis by modulating the neuroendocrine pathway, with the vagus nerve being a key part (Figure 2). The vagal pathway may be a potential therapeutic direction for the treatment of SAE by regulating gut flora.

### 3.3 Gut microbiota dysbiosis and neuroinflammation

Neuroinflammation is the main pathological process in SAE and is manifested by microglia activation, astrocyte proliferation, and infiltration of peripheral inflammatory mediators and immune cells (Gao Y. et al., 2022). During sepsis progression, specific pattern recognition receptors (PRPs) bind to pathogen-associated molecular patterns (PAMPs) of microorganisms and/or to danger-associated molecular patterns (DAMPs) of damaged tissues, stimulating the release of inflammatory mediators and amplifying the local inflammatory response (Hu et al., 2019). Inflammatory mediators modulate β-adrenergic, γ-aminobutyric acidergic or cholinergic neurotransmission and secretion of corticotropin-releasing factor, adrenocorticotropic hormone, and vasopressin, which affects neuroendocrine pathways, leads to severe systemic reactions, and consequently exacerbates SAE (Zhang et al., 2014). In addition, pro-inflammatory cytokines enter the central nervous system, thereby activating microglia (Lei et al., 2022). Activated microglia trigger a dual inflammatory pathway: releasing pro-inflammatory mediators (IFN-γ, IL-1β, TNFα, and ROS) to activate astrocytes, and producing chemokines to recruit leukocytes into the CNS. Both mechanisms synergistically exacerbate neuroinflammation, culminating in neuronal cell death, compromise of BBB integrity, and cerebral injury. Additionally, upregulated expression of inflammatory mediators within the microenvironment sustains microglial activation through autocrine signaling, establishing a self-reinforcing loop that amplifies neuroinflammatory damage (Lu J. et al., 2022; Parker et al., 2020; Ye et al., 2019).

The gut microbiota dysbiosis exacerbates sepsis-induced systemic inflammatory response. Intracerebral dissemination of polymicrobial organisms of intestinal origin has been detected in mice with experimental sepsis and in patients who died of sepsis, and the structure of the associated bacterial community is strongly correlated with the severity of neuroinflammation in SAE (Singer et al., 2018). Besides, gut microbiota dysbiosis stimulates the secretion of pro-inflammatory cytokines IL-1β, IL-6, and IL-18 by intestinal epithelial cells (IECs), intestinal dendritic cells, and macrophages. Gut microbiota and their constituent components (polysaccharide A and LPS) can interact with IECs Toll-like receptors (TLRs) to induce the production of cytokines such as TNF-α, IL-6, and IL-1, IL-12, and IL-10 (Li and Zhou, 2016; Zhao et al., 2018; Round and Mazmanian, 2010; Sun et al., 2020). The release of these inflammatory mediators into the body circulation induces a peripheral inflammatory infiltrate that drives a systemic inflammatory response and may further exacerbate SAE. Among them, TNF-α and IL-6 are the most important inflammatory factors in the early stage of sepsis: TNF-α causes neutrophil infiltration, brain tissue edema, and BBB dysfunction, and IL-6 indirectly mediates the hypothalamic-pituitary-adrenal axis by affecting the expression of cyclooxygenase 2 (COX2) and prostaglandin synthesis. This pro-inflammatory milieu leads to behavioral alterations, fever, and severe neurological impairments, which result in temporary and permanent cognitive deficits in survivors of sepsis, due to brain edema and neuronal apoptosis (Catarina et al., 2021; Huang et al., 2021). In addition, systemic attack by live bacteria, mainly Gram-negative *Escherichia coli* and *Salmonella typhimurium*, can cause microglia activation (Hoogland et al., 2015), and their main cell wall component, endotoxin, can bind to the TLRs on the surface of microglia, activate nuclear factor-κB (NF-κB), and cause inflammatory cascade responses that ultimately lead to neuroinflammation (Jamar et al., 2021; Zheng et al., 2021).

In summary, in sepsis models, gut microbiota dysbiosis leads to imbalance in the GBA immune pathways (Figure 2), resulting in activation of peripheral immune cells and release of inflammatory factors, exacerbating the systemic inflammatory response. At the same time, the integrity of the BBB is damaged, and inflammatory mediators can enter the brain and activate microglia and astrocytes, further promoting the development of neuroinflammation and ultimately leading to SAE.

### 3.4 Gut microbiota dysbiosis and metabolic dysfunction

Gut microbiota dysbiosis can be involved in the development of SAE by modulating metabolic pathways in the GBA axis. Studies have shown that *Sphingorhabdus* is negatively correlated with 2-ketobutyric acid, 9-decenoic acid, and L-leucine. It is positively correlated with glycylvaline. The *Eubacterium hallii* group is positively correlated with 2-methoxy-3-methylamine, acetaminophen, and synephrine acetonide. These correlations play a role in the development of SAE (Wang H. et al., 2022).

Gut microbiota may be involved in the pathophysiological process of SAE by regulating short-chain fatty acids (SCFAs), which are metabolites produced by the fermentation of indigestible dietary fiber by gut microflora (van der Hee and Wells, 2021). Acetate, propionate, and butyrate are the most abundant SCFAs in the human body (Martin-Gallausiaux et al., 2021). A significant decrease in the abundance of some beneficial bacteria positively associated with the production of SCFAs and cognitive function and an increase in the abundance of harmful bacteria associated with infection and cognitive dysfunction were found in a mouse model of sepsis, and cognitive dysfunction was significantly reversed in SAE mice after administration of SCFAs (Li et al., 2023). Decreased concentrations of acetate, propionate, and butyrate (major SCFAs) were found in experimental SAE models (Giridharan et al., 2022; Chen et al., 2021), and the KAT5 inhibitor NU9056 could achieve protective effects against BBB disruption and cognitive dysfunction in SAE mice by modulating the composition of the gut microbiota and up-regulating the concentrations of acetate, propionate, and butyrate (Chen et al., 2021). Based on this, it can be hypothesized that sepsis alters the composition and metabolism of the gut microbiota in mice and reduces the production of SCFAs, which is associated with cognitive impairment in SAE.

On the other hand, SCFAs also play an active role in SAE treatment. SCFAs can improve the integrity of the BBB by up-regulating the expression of ZO-1 and occludin to ameliorate the cognitive impairment in SAE (Li et al., 2023), and also reduce the over-activation of microglia and that of the production of pro-inflammatory cytokines IL-1β and IL-6, and decrease the levels of JNK, NF-κB, p65, and their phosphorylation levels in the mouse brain to achieve neuroprotective effects in SAE mice (Liu et al., 2021). In addition, butyrate partially activates GPR109A receptor on microglia, leading to activation of downstream Nrf2/HO-1 signaling pathway, thereby reducing oxidative stress response and neural lesion, ultimately alleviating the long-term cognitive impairment of SAE mice (Zhang et al., 2022). While these preclinical studies have yielded remarkable results, their translatability to human subjects requires further validation, particularly through multicenter, large-scale clinical trials.

Gut microbiota dysbiosis can also contribute to the development of SAE by affecting the balance of tryptophan metabolism. In the gut, the three main metabolic pathways of tryptophan [the production of indole derivatives, 5-HT, and kynurenine proton (Kyn)] are directly or indirectly controlled by the microbiota (Agus et al., 2018). Tryptophan can be metabolized by intestinal microorganisms to indoles and their derivatives such as indole-3-aldehyde (IAld), indole-3-acetic acid (IAA), and indole-3propionic acid (IPA). Tryptophan metabolites produced by commensal flora can control CNS inflammation through aryl hydrocarbon receptor (AhR)-mediated activation of microglia and transcriptional programs in astrocytes (Rothhammer et al., 2018). It was found that IPA was more enriched in the feces of SER mice than in SES mice, alleviated anxiety and spatial memory dysfunction in septic mice, significantly inhibited activation of NLRP3 inflammasome vesicles and IL-1β secretion in LPS-stimulated microglia, and that these effects were attenuated by antagonism of AhR. It suggests that IPA of gut microbial origin may be a potential therapeutic agent for the prevention of neuroinflammation in SAE (Fang et al., 2022).

In addition, approximately 90% of tryptophan is metabolized along the kynurenine pathway (Kennedy et al., 2017). Kynurenine can be converted into two distinct intermediates via two different pathways: the neurotoxic 3-hydroxykynurenine (3-HAA) and quinolinic acid (QA), and the neuroprotective kynurenic acid (KA) (Gao et al., 2016). Exploration of metabolic fingerprinting has revealed that L-kynurenine is progressively upregulated in sepsis and is strongly associated with the diagnosis and risk stratification of sepsis (Lu G. et al., 2022). CLP-induced sepsis results in markedly impaired hippocampus-dependent cognitive deficits, accompanied by increased kynurenine levels, elevated kynurenine/tryptophan ratios, reduced tryptophan expression, and decreased brain-derived neurotrophic factor (BDNF) concentrations. Single peripheral administration of the indoleamine-2,3-dioxygenase (IDO) metabolite L-kynurenine induces cognitive deficits similar to those caused by CLP, whereas the IDO inhibitor 1-methyl-D-tryptophan attenuates neuroinflammation by inhibiting pro-inflammatory cytokine release and kynurenine production, thereby protecting against sepsis-induced cognitive deficits in mice (Gao et al., 2016). Similarly, exogenous administration of kynurenic acid (KYNA) and its synthetic analogs (SZR-72 and SZR-104) exerts neuroprotective effects in experimental SAE by reducing peripheral neutrophil extracellular trap (NET) formation, attenuating BBB permeability alterations, and mitigating CNS mitochondrial dysfunction (Poles et al., 2021). Collectively, these findings highlight IDO-dependent neurotoxic kynurenine metabolism as a key contributor to sepsis-induced cognitive deficits and a potential target for SAE treatment.

Overall, sepsis disrupts gut microbiota composition, reduces SCFA-producing bacteria, and decreases systemic SCFA concentrations. These changes may aggravate intestinal and BBB disruption, thereby facilitating toxin translocation into the brain and promoting neuroinflammation, ultimately leading to SAE. Alterations in intestinal microbiota metabolism and their metabolites are involved in the etiology of SAE (Figure 2). Therefore, modulation of the gut microbiota and its metabolites may play a therapeutic role in SAE management.

### 3.5 Gut microbiota dysbiosis and neurotransmitter disorders

Gut microbiota synthesize and/or stimulate neurotransmitters, including gastrointestinal neurotransmitters such as γ-aminobutyric acid (GABA), 5-HT, norepinephrine, dopamine, acetylcholine, and histamine, via dietary amino acid metabolism (Snigdha et al., 2022). Gut microbiota dysbiosis leads to alterations in neurotransmitter levels. Decreased 5-HT levels in hippocampus, brainstem and frontal lobe were observed in mouse models of SAE induced by LPS and CLP (Zhang et al., 2024). Besides, an imbalance in the branched-chain to aromatic amino acid ratio occurs in early SAE, with elevated blood aromatic amino acids potentially increasing central nervous system uptake, leading to cerebral neurotransmitter dysfunction (e.g., impaired neurotransmitter synthesis or aberrant signaling) (Basler et al., 2002). Increased levels of the excitatory neurotransmitter glutamate (Glu) (Tang et al., 2023) and reduced BDNF (Gao L. et al., 2022) were observed in the hippocampus of experimental sepsis mouse models. Additionally, elevated Glu and reduced levels of the inhibitory neurotransmitter GABA were detected in SAE rats (Tang et al., 2023; Xi et al., 2021). Besides, late inflammation was associated with lower levels of BDNF and worse cognitive performance 30 days after sepsis (Biff et al., 2013). Administration of oral antimicrobials to SPF mice transiently altered the composition of the microbiota and increased exploratory behavior and hippocampal expression of BDNF (Bercik et al., 2011). Combined use of soybean embryo ethanol extract and *Lactobacillus gasseri* NK109 potently enhanced hippocampal BDNF expression, and the number of BDNF-positive neuron cells, while reducing LPS-induced cognitive impairment and colitis in mice (Biff et al., 2013; Yun et al., 2024).

In summary, gut microbiota dysbiosis exerts bidirectional effects on SAE pathogenesis. On the one hand, sepsis-induced alterations in gut microbiota composition and metabolites promote intestinal barrier and BBB permeability, trigger peripheral and central immune cell activation, and initiate inflammatory cascade responses. These processes further exacerbate barrier damage, forming a vicious cycle that drives immune cell infiltration and inflammatory mediator influx into the brain, ultimately leading to neuroinflammation and SAE development. Additionally, gut microbiota dysbiosis disrupts host metabolism and neurotransmitter homeostasis, contributing to SAE progression. Conversely, gut microbiota and their metabolites may exert protective effects by restoring intestinal barrier and BBB integrity, inhibiting immune activation, and reducing neuroinflammation.

However, there are differences in gut microbiota changes between septic rodents and septic patients. In a neuroinflammatory model of CLP-induced sepsis in rats, an upregulated Firmicutes/Bacteroidetes ratio in the gut was observed (Zhao et al., 2022), while the ratio was significantly lower on day 3 of sepsis diagnosis (Luan et al., 2024). Additionally, fundamental differences between rodents and humans include, but are not limited to, the divergence of the transcriptomic response, the mismatch of temporal response patterns, differences in both innate and adaptive immunity, and heterogeneity within the human population in comparison to the homogeneity of highly inbred mouse strains (Stortz et al., 2017). These factors may lead to the conclusion that gut microbiota dysbiosis promotes the development of SAE not being fully applicable to humans. While some correlative studies can be performed in human sepsis, the initial testing of pharmacological agents and determination of the mechanistic basis of their action are not possible with human subjects. Therefore, future efforts could focus on developing new preclinical animal models [e.g., humanized mice and human organoid mice (Cai et al., 2023)] to facilitate clinical translation.

## 4 Potential therapeutic interventions for SAE targeting the gut microbiota

In the human body, the gut microbiota is dynamic and diverse, and external interventions can be applied to modulate its composition and function. Therefore, the gut microbiota represents a promising therapeutic target for SAE. Emerging evidence supports the efficacy of microbiota-targeted therapies, including FMT and probiotics, in ameliorating SAE outcomes.

### 4.1 Fecal microbiota transplantation

Fecal microbiota transplantation involves transferring a healthy gut microbiota from a donor to a patient’s gastrointestinal tract to restore microbial homeostasis and improve clinical outcomes (Dodiya et al., 2022). Preclinical studies suggest FMT may be effective in ameliorating sepsis and SAE symptoms. FMT reduces morbidity and mortality in septic mice while restoring gut microbiota abundance and diversity. It improves intestinal barrier function by downregulating epithelial cell apoptosis, enhancing mucus layer composition, upregulating TJ proteins (e.g., ZO-1 and occludin), and reducing intestinal permeability and inflammation (Gai et al., 2021). Additionally, FMT regulates gut microbiota dysbiosis, activates CAPs, and ameliorates cognitive dysfunction in septic rats (Li et al., 2019). In SAE rats, FMT alleviates hippocampal injury by inhibiting inflammatory cytokine secretion, reducing IBA-1 expression, correcting neurotransmitter imbalances, and suppressing M1 macrophage polarization in mesenteric lymph nodes (MLNs) (Xi et al., 2021). Comparative studies show FMT outperforms prebiotics, probiotics, and synbiotics in restoring gut microbiota composition and improving cognitive function in septic rats (Li et al., 2021). These findings demonstrate that FMT has shown positive outcomes in correcting intestinal dysbiosis and improving SAE animals. However, in clinical disease models, there are still gaps in the treatment of SAE with FMT, which can be further explored.

In recent years, the concept of whole intestinal microbiota transplantation (WIMT) has been put forward. WIMT involves transferring microbiota from the jejunum, ileum, cecum, and colon, introducing more small intestine-derived microorganisms and related microbial functions into the recipient’s intestine. Compared with FMT, it can remodel the entire gut microbiota, improves intestinal morphology, and reduces systemic inflammation in recipients (Li et al., 2020). In DSS-induced IBD mice, WIMT demonstrates superior therapeutic effects compared to FMT, which may be attributable to the enrichment of metabolic pathways involving SCFAs and *Bifidobacterium* (Yang et al., 2023). Given these advantages, future studies should investigate the therapeutic potential of WIMT in SAE. Additionally, with the continuous deepening of research and the advancement of technology, metagenomics sequencing-based establishment of fecal biobanks and targeted infusion of fecal components (Bacteria, Virome, or bacteriophage) (Yu et al., 2023; Wu et al., 2023) hold enormous therapeutic potential.

### 4.2 Probiotics

Probiotics are defined as “live microorganisms that, when administered in adequate amounts, confer health benefits to the host” (Hill et al., 2014). This definition emphasizes their microbial nature, viability, and health-promoting properties. Preclinical studies demonstrate that prophylactic administration of *Lactobacillus rhamnosus* GG (LGG) protects against sepsis in rats (Yılmaz and Erdem, 2020). LGG supplementation significantly alleviated sepsis-caused decreases in hippocampal BDNF expression and p-TrkB phosphorylation levels, preserving neuronal survival and improving cognitive impairments in mice with sepsis (Wang et al., 2024b). A 4-week continuous gavage of LGG reversed gut microbiota dysbiosis in septic mice, reducing potentially pathogenic bacteria and increasing beneficial species, while rebalancing lipid and bile acid metabolism. These effects restored intestinal barrier function, attenuated inflammation, and reduced mortality (Chen et al., 2020). A randomized trial showed that multispecies probiotics restored gut microbiota composition in early sepsis, enriching *Lactobacillus* and enhancing microbial functional diversity (Stadlbauer et al., 2019). Mechanistically, probiotics alleviate SAE-related brain dysfunction by inhibiting pro-inflammatory cytokines (e.g., TNF-α and IL-6) through microbiota modulation. In a mouse model, 1-month administration of probiotics containing *Clostridium butyricum* (Cb) improved cognitive function, reduced neuronal damage, suppressed neuroinflammation, and increased BDNF levels (Liu et al., 2020). Collectively, probiotics represent a promising therapeutic strategy for SAE by restoring gut microbiota and metabolic homeostasis, improving cognitive outcomes, and reducing sepsis mortality. However, a RCT of the probiotic *Bifidobacterium breve* as prophylaxis in over 1,000 very preterm infants at high risk of sepsis did not demonstrate reduction in sepsis incidence or mortality compared to placebo (Costeloe et al., 2016). Meanwhile, concurrent broad-spectrum antibiotic use in patients with sepsis may limit probiotic colonization and beneficial effects (Manzanares et al., 2016). Therefore, the clinical application of probiotics should be comprehensively judged by integrating patients’ individual characteristics, disease types, and strain-specific properties.

### 4.3 Challenges and limitations

Despite the promising results of microbiota-targeted therapies in preclinical models, their clinical application remains ambiguous with mixed prospects. Currently, FMT has achieved remarkable efficacy in treating various gastrointestinal diseases (Wang et al., 2024a; Costello et al., 2019), and has also demonstrated therapeutic potential in neurological disorders such as Parkinson’s disease (Cheng et al., 2023; Bruggeman et al., 2024), amyotrophic lateral sclerosis (Feng et al., 2024), and autism spectrum disorder (Kang et al., 2020). However, a significant limitation of current research is the lack of standardized FMT treatment protocols, including donor/recipient screening, infusion volume and frequency of fecal matter, administration routes and timing, and whether to combine with other drug therapies (Porcari et al., 2023; Ooijevaar et al., 2019; Carlson, 2020). Among them, the efficacy and safety of FMT are highly dependent on donor screening. Despite the critical importance of donor screening in FMT, identifying and recruiting appropriate donors remains fraught with challenges. First, high-quality donor screening protocols are associated with substantial financial costs (Bénard et al., 2022). In a Dutch cohort, disqualification of potential donors often resulted from the presence of protozoa *Dientamoeba fragilis* and *Blastocystis* spp. (Bénard et al., 2022). Moreover, the elevated donor attrition rate is predominantly driven by the onerous stool sample donation protocol, behavioral limitations imposed on donors, and substantial time obligations (Cammarota et al., 2019; McSweeney et al., 2020). Additionally, the safety of FMT in clinical application requires further clarification. A global analysis of FMT safety showed that the most common short-term FMT-related adverse events are diarrhea, abdominal discomfort/pain/cramping, with severe cases involving infection or death (Wang Y. et al., 2022). Another real-world data shows that the risk of developing new medical conditions beyond 12 months after FMT is low (Yau et al., 2024). However, FMT may cause the transmission and clearance of potential carcinogenic bacteria. Four of the 11 patients demonstrated potential engraftment after FMT of donor strains harboring virulence factors (Drewes et al., 2019). This suggests that further studies on appropriate screening measures for FMT donors and the long-term consequences and/or benefits of FMT are warranted.

Although probiotics have shown certain potential in clinical treatment and health maintenance, their practical application is subject to numerous limitations. First, empirical probiotic supplementation may be limited by mucosal colonization resistance (Zmora et al., 2018). Second, there is currently no consensus on optimal probiotic strains, dosages, and treatment durations. Moreover, probiotics have not been proven to have long-term beneficial effects, are only effective under certain conditions and may even cause side effects. *C. butyricum*, a probiotic commonly prescribed in Asia, occasionally leads to bacteremia (Sada et al., 2024; Jiang et al., 2025), but the prevalence and characteristics of C. *butyricum* bacteremia and its bacteriologic and genetic underpinnings remain unknown (Sada et al., 2024). Additionally, a markedly higher risk of *Lactobacillus* bacteremia for ICU patients treated with probiotics compared to those not treated (Yelin et al., 2019). Therefore, the selection of probiotics should be based on individualized assessment, combined with treatment objectives, while avoiding the abuse of probiotics.

## 5 Conclusion

Sepsis-associated encephalopathy is one of the most common critical encephalopathies, with disease severity closely linked to patient prognosis and quality of life. Current evidence indicates that sepsis-induced gut microbiota dysbiosis contributes to SAE pathogenesis by disrupting intestinal barrier and BBB integrity, amplifying systemic inflammation and neuroinflammation, and promoting metabolic and neurotransmitter imbalances. This mechanism provides novel therapeutic targets for SAE. Microbiota-based therapies, including FMT and probiotics, play a crucial role in ameliorating SAE symptoms and improving patient outcomes. However, optimal criteria for treatment timing, dosage, and strain selection remain unclear. Future research should focus on elucidating the gut microbiota’s contribution to SAE development, with in-depth investigations into gut microbial metabolites offering potential insights into pathogenesis and therapeutic innovation.
